# Macronutrients modulate survival to infection and immunity in *Drosophila*

**DOI:** 10.1111/1365-2656.13126

**Published:** 2019-12-09

**Authors:** Fleur Ponton, Juliano Morimoto, Katie Robinson, Sheemal S. Kumar, Sheena C. Cotter, Kenneth Wilson, Stephen J. Simpson

**Affiliations:** ^1^ Department of Biological Sciences Macquarie University Sydney NSW Australia; ^2^ Charles Perkins Centre and School of Life and Environmental Sciences The University of Sydney Sydney NSW Australia; ^3^ School of Life Sciences University of Lincoln Lincoln UK; ^4^ Lancaster Environment Centre Lancaster University Lancaster UK

**Keywords:** *Drosophila*, immunity, infection, macronutrients, nutrition

## Abstract

1. Immunity and nutrition are two essential modulators of individual fitness. However, while the implications of immune function and nutrition on an individual's lifespan and reproduction are well established, the interplay between feeding behaviour, infection and immune function remains poorly understood. Asking how ecological and physiological factors affect immune responses and resistance to infections is a central theme of eco‐immunology.
2. In this study, we used the fruit fly, *Drosophila melanogaster*, to investigate how infection through septic injury modulates nutritional intake and how macronutrient balance affects survival to infection by the pathogenic Gram‐positive bacterium *Micrococcus luteus*.
3. Our results show that infected flies maintain carbohydrate intake, but reduce protein intake, thereby shifting from a protein‐to‐carbohydrate (P:C) ratio of ~1:4 to ~1:10 relative to non‐infected and sham‐infected flies. Strikingly, the proportion of flies dying after *M. luteus* infection was significantly lower when flies were fed a low‐P high‐C diet, revealing that flies shift their macronutrient intake as means of nutritional self‐medication against bacterial infection.
4. These results are likely due to the effects of the macronutrient balance on the regulation of the constitutive expression of innate immune genes, as a low‐P high‐C diet was linked to an upregulation in the expression of key antimicrobial peptides.
5. Together, our results reveal the intricate relationship between macronutrient intake and resistance to infection and integrate the molecular cross‐talk between metabolic and immune pathways into the framework of nutritional immunology.

## Graphical abstract

Self‐medication has been traditionally seen as animals using molecules such as secondary plant compounds or other non‐nutritive substances with antiparasitic activity. The authors' results support that self‐medication can happen through modulating dietary macronutrient selection to stimulate immunity and compensate for the negative effects of the infection on fitness traits.

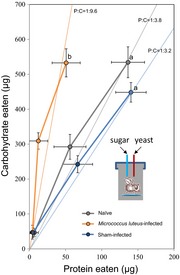

## 1 INTRODUCTION

In nature, most individuals will be exposed to parasites at least once in their lifetime with important consequences for the expression of their life‐history traits as well as the structure of populations and ecosystems (Schmid‐Hempel, 2011). Because of the constant evolutionary pressure from parasites, hosts have evolved immune defences to eliminate and/or mitigate the burden of parasitic infection, while parasites have evolved strategies to evade hosts' immune adaptations (generating an ‘evolutionary arms race’). Gaining a better understanding of how ecological factors and behavioural responses affect host immune responses and parasite susceptibility is a central topic in the field of eco‐immunology (Schmid‐Hempel, 2011).

Nutrition is a key ecological factor modulating the expression of life‐history traits (Simpson & Raubenheimer, 2012) and the response of hosts to infection (Amar, Zhou, Shaik‐Dasthagirisaheb, & Leeman, 2007; Ayres & Schneider, 2009; Bauer et al., 2006; Calder, 2006; Cunningham‐Rundles, McNeeley, & Moon, 2005; Falagas, Athanasoulia, Peppas, & Karageorgopoulos, 2009; Falagas & Kompoti, 2006; Genoni et al., 2014; Hawley & Altizer, 2010; Huttunen & Syrjanen, 2013; Kelley & Bendich, 1996; Klasing, 2007; Lazzaro & Little, 2009; Martinez et al., 2014; Ponton, Wilson, Cotter, Raubenheimer, & Simpson, 2011; Ponton et al., 2013; Rolff & Siva‐Jothy, 2003; Samartin & Chandra, 2000; Schmid‐Hempel, 2011; Sheldon & Verhulst, 1996; Sorci & Faivre, 2009; Vogelweith, Moreau, Thiery, & Moret, 2015; Wu, Randle, & Wu, 2007). Recent studies have allowed a detailed molecular understanding of the cross‐regulation between nutrition and immunity, with nutrient sensing pathways being identified as important regulators of innate immunity (Becker et al., 2010; Martin, Saha, & Riley, 2012; Varma, Bülow, Pesch, Loch, & Hoch, 2014). Immune responses can be activated independently of an infection, and this regulation can be modulated by the availability of nutrients (see, for instance, Vogelweith et al., 2015). While the underlying mechanisms are far from being fully understood, the relationship between diet, diet‐induced metabolic diseases and infections is clearly multifactorial, with impairments of immune function playing a key role (Martí, Marcos, & Martínez, 2001; Nave, Beutel, & Kielstein, 2011). Therefore, gaining a better understanding of the nutritional components that influence immunity and resistance to infection is an important challenge, with broad implications across health, nutrition, ecological and organismal science.

There is an ongoing debate on the effects of diet on immune responses to infections. Food deprivation and/or protein shortage have been reported to negatively affect immune responses and survival after infection (Brunner, Schmid‐Hempel, & Barribeau, 2014; Pletcher et al., 2002; Siva‐Jothy & Thompson, 2002; Tritschler et al., 2017; Wilson et al., 2019) with infected hosts generally selecting a protein‐biased diet that provides them with a better survival after infection (Lee, Cory, Wilson, Raubenheimer, & Simpson, 2006; Povey, Cotter, Simpson, Lee, & Wilson, 2009; Povey, Cotter, Simpson, & Wilson, 2014). In *Drosophila*, while diet restriction has been shown to decrease the capacity of the host to clear the infection (i.e. ‘resistance’), it provided the host with the ability to reduce the damage of the infection on its health, also called ‘tolerance’ (Ayres & Schneider, 2009, 2012). Diet composition may affect tolerance of infection (Howick & Lazzaro, 2014; Kutzer & Armitage, 2016; Miller & Cotter, 2017); for example, it has been shown that yeast restriction in *Drosophila* flies affects tolerance specifically to one strain of bacterium in a time‐dependent manner; however, no effect on resistance was detected (Kutzer & Armitage, 2016).

Finally, a number of recent studies have revealed a negative effect of protein and/or a positive effect of carbohydrate on resistance (Graham et al., 2014; Kay et al., 2014; Mason, Smilanich, & Singer, 2014) with, for instance, female *Drosophila* fed an holidic diet supplemented with glucose having greater survival following infection with the gut pathogen *Vibrio cholerae* (Galenza, Hutchinson, Campbell, Hazes, & Foley, 2016). Although there is a clear effect of diet composition on resistance to infection and immune state, dietary manipulations have usually focused on changing single nutrients or varying the caloric content and nutrient ratio simultaneously, which hinders the ability to specifically measure the effects of food components and/or caloric content on immunity (but see Cotter et al., 2019; Cotter, Simpson, Raubenheimer, & Wilson, 2011). There is now growing evidence that considering the interactive effects of nutrients is essential and offers a more ecologically relevant understanding (Cotter et al., 2019; Cotter, Simpson, et al., 2011; Simpson, Couteur, & Raubenheimer, 2015; Simpson & Raubenheimer, 2012).

How the nutritional requirements of an organism, its foraging behaviour and metabolism interact and are linked to the environment is central question of nutritional ecology, as nutrition links individuals, populations, communities and ecosystems. Here, we explore the nutritional responses of *Drosophila melanogaster* after bacterial challenge and the consequences of such responses for survival following infection. We performed a detailed investigation of the dietary modulation of constitutive innate immune gene expression in an age‐dependent manner. The effects of nutrition were measured through a geometric manipulation of the dietary protein and carbohydrate balance. Our observations unveiled nutritional regulations of innate immune gene expression and resistance to bacterial infections and link these findings to nutritional self‐medication.

## 2 MATERIALS AND METHODS

### 2.1 Experimental infection

One‐day‐old adult female flies (Canton‐S, stock from Bloomington) were experimentally infected using a solution of freshly grown *Micrococcus luteus* (ATCC 10240) at OD_600_ = 0.5. Flies were anaesthetized under CO_2_ and pricked in the thorax using a dissecting pin that was beforehand dipped in the bacterial solution [see Apidianakis & Rahme, 2009]. We also generated sham‐infected flies using a pin dipped in ethanol (70%). As negative controls, we used non‐infected, non‐injured flies (i.e. naïve flies). Flies were left to recover from pricking for half an hour. Survival immediately after the infection was ~95%.

### 2.2 Nutritional intake target

Infected, sham‐infected and naïve flies were individually provided with two 5‐µl microcapillary tubes (Drummond Microcaps) filled with liquid diets (*n* = 20 flies per treatment at the start of the experiment): one diet consisted of autolysed yeast (MP Biomedicals, catalogue no. 103304) at 180 g/L and the other of sucrose at 180 g/L. The two solutions were prepared in sterile, distilled water. Intake was measured against a scale bar by height difference in the column of liquid within the microcapillary every 2 days for 6 days (see Lee et al., 2008; Ponton et al., 2015). Total quantities of protein and carbohydrate ingested were compared using one‐way ANOVA type II and post hoc tests (Tukey's HSD).

### 2.3 Effect of dietary manipulation on resistance to infection

One‐day‐old adult female flies were infected as described above. Flies were left to recover for half an hour before being transferred to experimental cages and split into groups of 50 individuals fed with three solid diets varying in the P:C ratio. Foods varied in autolysed yeast (Y) and sucrose (S) content. The Y:S concentration was 180 g/L. Macronutrient compositions were calculated based on autolysed yeast [MP Biomedicals, catalogue no. 103304 containing 62% protein]. Each diet contained 0.01% phosphoric acid and 0.1% propionic acid as antimould agents and were prepared in sterile, distilled water. Dietary treatments were defined as ‘high P:C ratio’ (i.e. P:C = 1:1 or 52% P), ‘medium P:C ratio’ (i.e. P:C = 1:4 or 24% P) and ‘low P:C ratio’ (i.e. P:C = 1:32 or 4% P). The medium ratio represents the preferred choice of healthy flies and one that maximizes lifetime reproductive success (Lee et al., 2006). Three replicate cages for *M. luteus*‐ and sham‐infected flies and two replicate cages with naïve flies were run in parallel for each dietary treatment. Lifespan was followed for 16 days with dead flies counted daily. Flies that died from 0 to 6 hr post‐infection were removed from the analyses since we could not assess whether the death was directly caused by the infection or the dietary treatment. Kaplan–Meier lifespan curves were analysed using Cox regression and log‐rank Mantel–Cox tests.

### 2.4 Immune gene expression levels using RT‐qPCR

We investigated the expression of immune genes of the IMD and Toll pathways using reverse transcription quantitative PCR (RT‐qPCR). We used 1‐day‐old adult female infected, sham‐infected or control flies. After pricking, flies were left to recover for half an hour before being transferred to P:C = 1:4 (3 replicate cages per treatment). After 6 hr, flies were dissected (i.e. eggs removed), preserved in RNA later (Ambion) and stored at −80°C. RNA was extracted for 10–15 flies per cage (see below for more details on RNA extraction). Complementary DNA was generated using the QuantiTect Reverse Transcription Kit (Qiagen). Triplicate cDNA aliquots for each sample served as templates for quantitative PCR using SYBR Green PCR Master Mix (Applied Biosystems). Amplification reactions were performed in 10 µl total volumes with 4.5 μl of cDNA (diluted 1:90) and 100–200 nM of each primer [see Ponton, Chapuis, Pernice, Sword, and Simpson (2011) for the primer sequences of reference genes Rpl32 (ribosomal protein l32, CG7939) and Ef1 (elongation factor 1, CG1873); see Table S1 for the primer sequences of target genes], in 384‐well optical plates under the following sequential conditions: incubation at 50°C for 2 min, 95°C for 10 min, followed by 45 cycles of 95°C for 15 s and 60°C for 1 min. RT‐qPCR efficiency was determined for each gene and each treatment using the second derivative method. Relative standard curves for the gene transcripts were generated with serial (5×) dilutions of cDNA (i.e. 1/20, 1/40, 1/80, 1/160 and 1/320). Stock cDNA used for the relative standard curves consisted of a pool of cDNA from the different samples. No template (to check for contamination of chemicals) and no reverse transcriptase (i.e. no RT, to check for genomic DNA amplification) controls were run for each primer pair. Target gene expression levels were normalized by reference gene expression levels. Expression levels were given relative to the control treatment (i.e. non‐injured, non‐infected flies) for each gene and compared between treatments using one‐way ANOVAs followed by post hoc tests (Tukey's HSD).

### 2.5 Immune gene expression levels using TaqMan low‐density array (TLDA) cards

#### 2.5.1 Dietary treatments

Foods varied in autolysed yeast (Y) and sucrose (S) content. The seven Y:S ratios used were 1:14, 1:7, 1:3.5, 1:1.6, 1:0.7, 1:0.2 or 1:0; yielding protein‐to‐carbohydrate ratios of 1:21, 1:11, 1:5, 1:2.5, 1:1, 3:1 and 1:0, respectively; and percentages of protein (w/w(Y + S)) of 4%, 8%, 14%, 24%, 36%, 52% and 62%, respectively. The Y + S concentration was 180 g/1. Macronutrient compositions were calculated based on autolysed yeast [MP Biomedicals, catalogue no. 103304 containing 62% protein]. Each solid diet contained 0.01% phosphoric acid and 0.1% propionic acid as antimould agents and was prepared in sterile, distilled water.

#### 2.5.2 Fly sampling

Newly eclosed female flies were sorted and placed in longevity cages. Three replicate cages were run per diet (i.e. P:C 1:21, 1:11, 1:5, 1:2.5, 1:1, 3:1 and 1:0), each with 180 flies. Dead individuals were counted and removed from the cages every 2 days until all flies were dead. Life expectancy curves were analysed using log‐rank Mantel–Cox tests. Ten live flies per treatment cage were sampled at 25%, 50% and 75% mortality (see Figure S1 and Table S2). Flies were therefore sampled at 3 similar ‘physiological ages’ and not at a ‘fixed age’ since flies do not age at the same rate on the different diets. Flies were dissected (i.e. eggs removed), preserved in RNA later (Ambion) and stored at −80°C for further analyses.

#### 2.5.3 RNA extractions

We prepared up to three total RNA samples per dietary treatment by pooling 10 individuals per replicate cage per treatment. When less than 10 flies remained in the longevity cages, we discarded the sample. Subsequently, total RNA was extracted using a Trizol/RNeasy (Plus Mini kit, Qiagen) hybrid extraction protocol [see Ponton, Chapuis, et al. (2011)]. Briefly, insects were homogenized in 1 ml TRIzol reagent using a TissueLyser and 7‐mm stainless beads. Samples were incubated for 15 min at room temperature and centrifuged for 10 min at 12,000 *g* at 4°C. A standard volume of supernatant (800 μl) was removed and added to 200 μl of chloroform. Tubes were shaken vigorously for 15 s, incubated at room temperature for 3 min and centrifuged for 20 min at 12,000 *g* at 4°C. The aqueous phase (350 μl) was transferred to a gDNA eliminator column from an RNeasy Plus Mini Kit (Qiagen), and all other steps were performed according to the manufacturer's protocol (i.e. from step 4 in the version from Oct. 2005). Total RNA was eluted in 35 μl of water. Extraction was followed by a DNase treatment (Ambion) to eliminate potential genomic DNA in the samples. RNA was then stored at −80°C before further processing. The quality and quantity of RNA were assessed with a Nanodrop ND‐1000 spectrophotometer (Nanodrop Technologies). cDNA was produced using the QuantiTect Reverse Transcription Kit (Qiagen). cDNA was stored at −20°C until used.

#### 2.5.4 Gene expression analysis

Gene expression was evaluated using custom‐made TaqMan low‐density array (TLDA) cards (Life Technologies/Applied Biosystems). Each TLDA card allowed for eight samples and assayed the expression of 21 immune genes (see Table S3). Target gene expression levels were normalized using four reference genes (i.e. Ef1α100E, αTub84B, RpL32 and 18SrNA, see Table S3). All samples were run on an ABI model 7900HT sequence detection system according to the protocol supplied by the manufacturer. Results were summarized using the 2^−∆∆Ct^ method. We log‐transformed the response variable before making statistical inferences, although all plots are of the raw data.

The effect of the percentage of dietary P:C was first tested on all genes and genes classified per function using Kruskal–Wallis tests. The effect of the percentage of dietary P:C was then tested for each gene and time point individually using generalized additive models (GAMs) that allowed for no a priori decision for choosing a particular response function. The percentage of protein in the diet was used as a descriptor of the diet composition.

### 2.6 Statistical analyses

Statistical analyses were run using r (R Core Team, 2014) and spss (IBM Corp. released 2012. IBM SPSS Statistics for WINDOWS, v. 21.0; Armonk, NY: IBM Corp.).

## 3 RESULTS

### 3.1 Bacterial infection induces a shift in dietary choice to a low P:C diet

**Figure 1:**
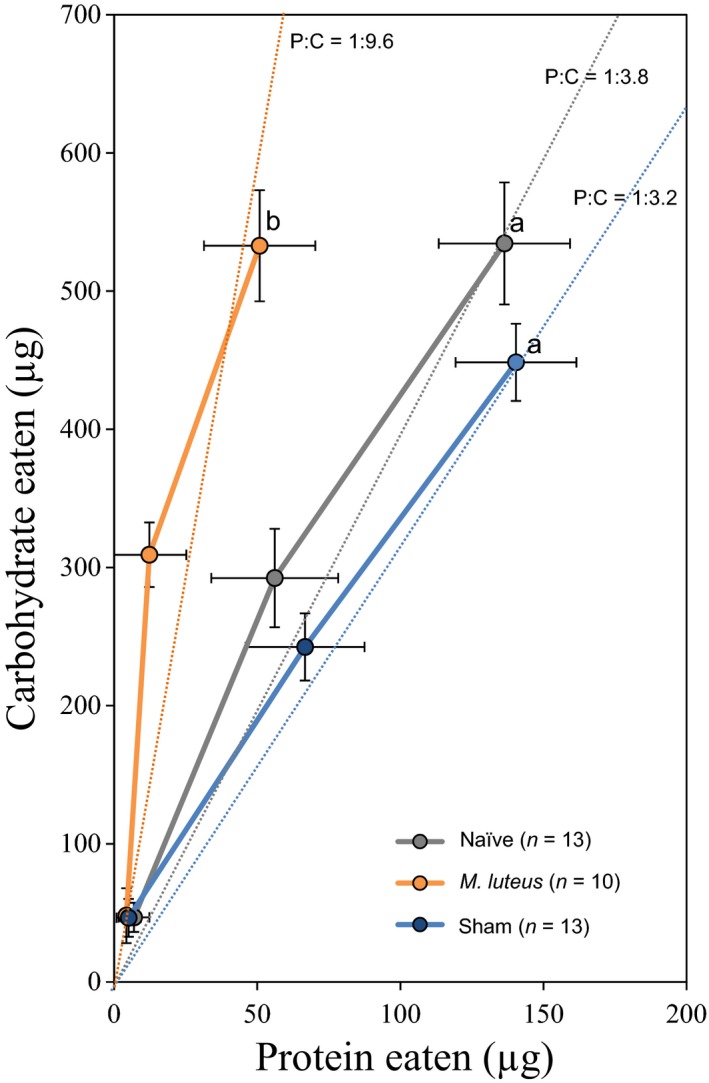
Cumulative protein–carbohydrate intake (mean ± *SE*) trajectories at 2‐day intervals over 6 days. Dotted lines represent protein‐to‐carbohydrate ratios (P:C). Letters indicate significant HSD Tukey's pairwise comparisons (*p* ≤ .05)

We first hypothesized that infection through septic injury with the pathogen *M. luteus* would modulate the nutritional selection of *D. melanogaster*. Adult flies were offered a choice between two capillaries filled with either a sucrose or a yeast solution, and food intake was measured every 2 days for 6 days (Ja et al., 2007). While non‐, sham‐ and *M. luteus*‐infected flies ingested similar quantities of carbohydrate (cumulative consumption of carbohydrate over 6 days, one‐way ANOVA, *F*_2,36_ = 1.775, *p* = .185, Table S4), flies infected with *M. luteus* ate significantly less protein than sham‐infected or non‐infected flies (i.e. cumulative consumption of protein for 6 days, one‐way ANOVA, *F*_2,36_ = 5.853, *p* = .007, Table S4; Figure 1). This reduction in protein intake by infected flies resulted in a marked change in the ingested dietary P:C ratio, such that flies infected with *M. luteus* balanced their diet to a P:C ratio close to 1:9.6 (i.e. 9% protein, Figure 1) and non‐ and sham‐infected to a P:C ratio of 1:3.8 (i.e. 20% protein) and 1:3.2 (i.e. 25% protein), respectively (Figure 1).

These first results show that when flies are infected with *M. luteus*, they shift their nutritional choice to a carbohydrate‐biased (lower P:C) diet, which is above and beyond the stress of physical injury (i.e. compare sham‐infected vs. infected).

### 3.2 A low P:C diet can improve survival post‐infection

**Figure 2:**
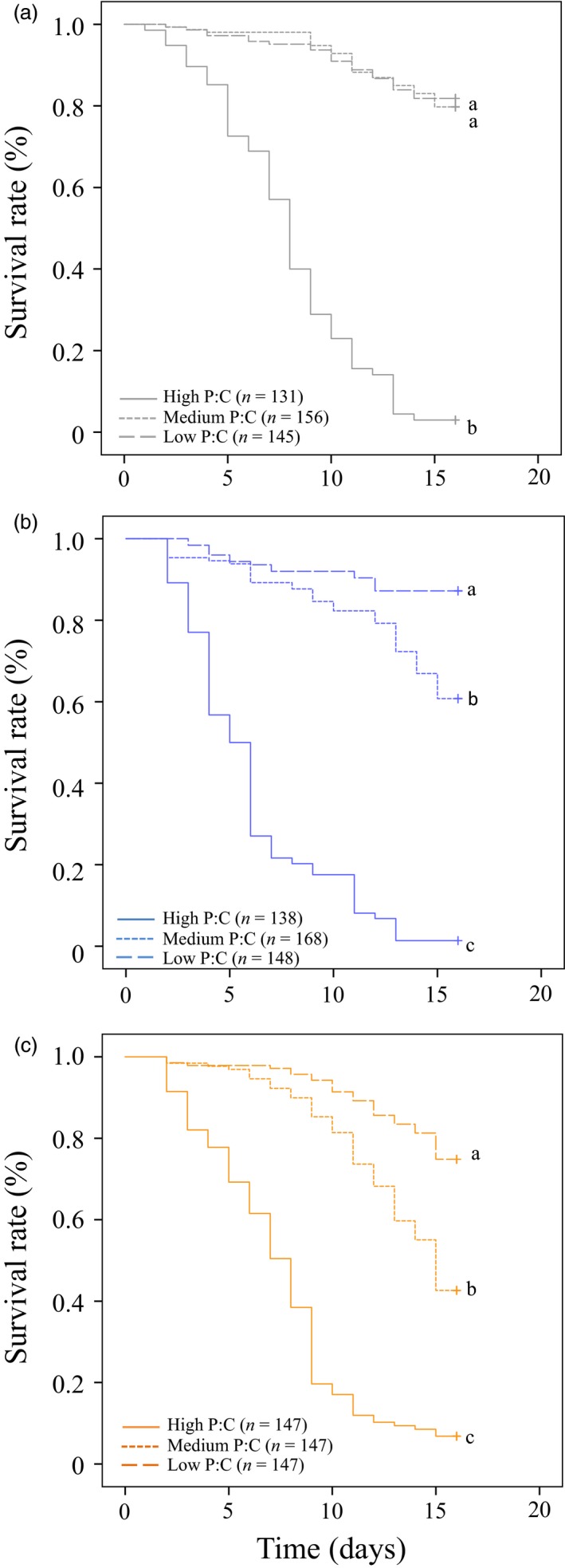
Survival curves of flies fed one of three diets varying in the protein‐to‐carbohydrate ratio (P:C) [i.e. P:C = 1:1 (high), 1:4 (medium) or 1:32 (low)] after treatment [naïve (a), sham‐ (b) and *Micrococcus luteus*‐infected (c)]. Letters indicate significant pairwise comparisons (*p* ≤ .05)

We then hypothesized that the shift to a low P:C diet observed for infected flies had survival value. In this second experiment, non‐, sham‐ and *M. luteus*‐infected flies were fed one of three diets (high, medium and low P:C in a no‐choice experiment) and survival was followed. As expected, the interaction between the dietary P:C and the treatment significantly influenced survival rates of flies (Cox regression, Treatment X Diet: *χ* ^2^ = 26.97, *df* = 4, *p* < .001, Treatment: *χ* ^2^ = 66.28, *df* = 2, *p* < .001, Diet: *χ* ^2^ = 606.57, *df* = 2, *p* < .001). Survival was reduced on higher P:C diets for all three groups of flies compared to the two other diets (Figure 2). However, while naïve flies survived in similar proportions on medium and low P:C diets (i.e. 24% and 4% protein) (log rank pairwise comparisons, *p* > .05, Figure 2a), *M. luteus*‐ and sham‐infected flies survived significantly better on the low P:C diet (i.e. 4% protein) compared to the medium P:C diet (i.e. 24% protein diet) (log rank pairwise comparisons, *p* ≤ .05; Figure 2b,c). The interaction between diet and treatment significantly influenced the percentage of dead flies at day 15 (Table S5A). Flies on the high P:C diet had a greater percentage of death (Table S5B; post hoc test, *p* > .05). On the medium P:C diet, we found greater mortality for flies infected with *M. luteus* compared to sham‐infected and naïve treatments (Table S5B; post hoc test, *p* ≤ .05). Mortality was also greater for sham‐infected flies compared to naïve individuals. On the low P:C diet, however, the percentage of dead flies was not significantly different between the three treatments (Table S5B; post hoc test, *p* > .05). These results suggest that infected flies can improve their survival by shifting to a low‐protein, high‐carbohydrate diet.

### 3.3 The P:C ratio influences the constitutive expression of antimicrobials

**Figure 3:**
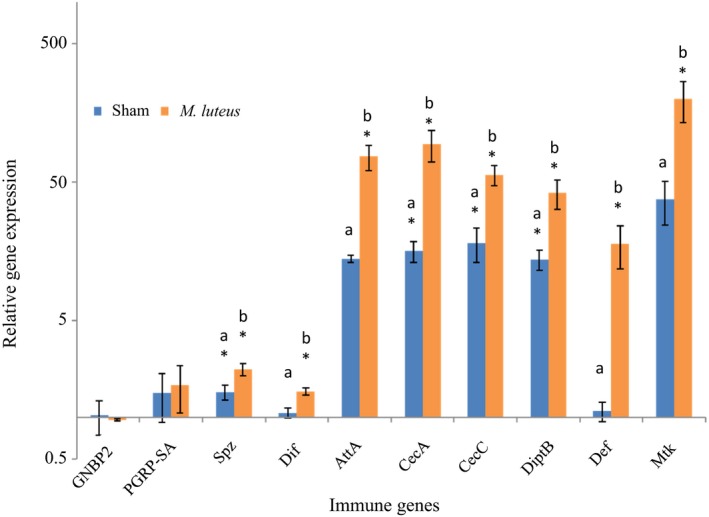
Relative immune gene expression (relative to naïve flies, mean + *SEM*). Letters indicate significant HSD Tukey's pairwise comparisons (*p* ≤ .05) between sham‐injured and *Micrococcus luteus‐*infected flies; stars (*) indicate significant Bonferroni pairwise comparisons (*p* ≤ .05) against naïve flies (see also Table S6)

We next investigated the underlying mechanisms mediating the effects of a carbohydrate‐biased diet on the immune state. We first checked whether the bacterial‐ and sham‐infection treatments influenced the expression of immune genes 6 hr post‐challenge. We hypothesized that infection with *M. luteus* stimulates expression of AMPs and genes involved in the transduction of the immune signal in a greater way than sham‐injection (i.e. injury). Infection with *M. luteus* triggered the enhanced expression of all of the antimicrobial peptides assayed (i.e. AttaA, CecA, CecC, DipB, Def, Mtk, Table S6 and Figure 3), as well as molecules involved in the transduction of the immune signal (i.e. spz and Dif, Table S6 and Figure 3). Relative to naïve flies, however, no significant effect of infection was detected on the level of expression of the two receptors involved in the recognition of pathogens we measured (i.e. GNBP2 and PGRPSA, Table S6 and Figure 3). Even though the expression levels of spz, CecA, CecC and DiptB were significantly greater in sham‐infected flies compared to non‐infected insects, levels of expression of these genes remained more elevated in infected individuals compared to sham‐infected and non‐infected individuals (Figure 3).

We hypothesized that a low‐protein, high‐carbohydrate diet stimulates the expression of immune genes. To test this, we measured the expression of 21 genes involved in the integrated response to pathogen infection, beginning with pathogen recognition receptors, transduction of the immune signals and antimicrobial peptides (AMPs) for flies fed seven isocaloric diets varying in the P:C ratio (the percentage of dietary protein was used as a descriptor in the analyses and figures, see Methods and Table S3). Flies were sampled at three key points on their life expectancy curves (i.e. 25%, 50%, 75% mortality, see Figure S1 and Table S2).

**Figure 4:**
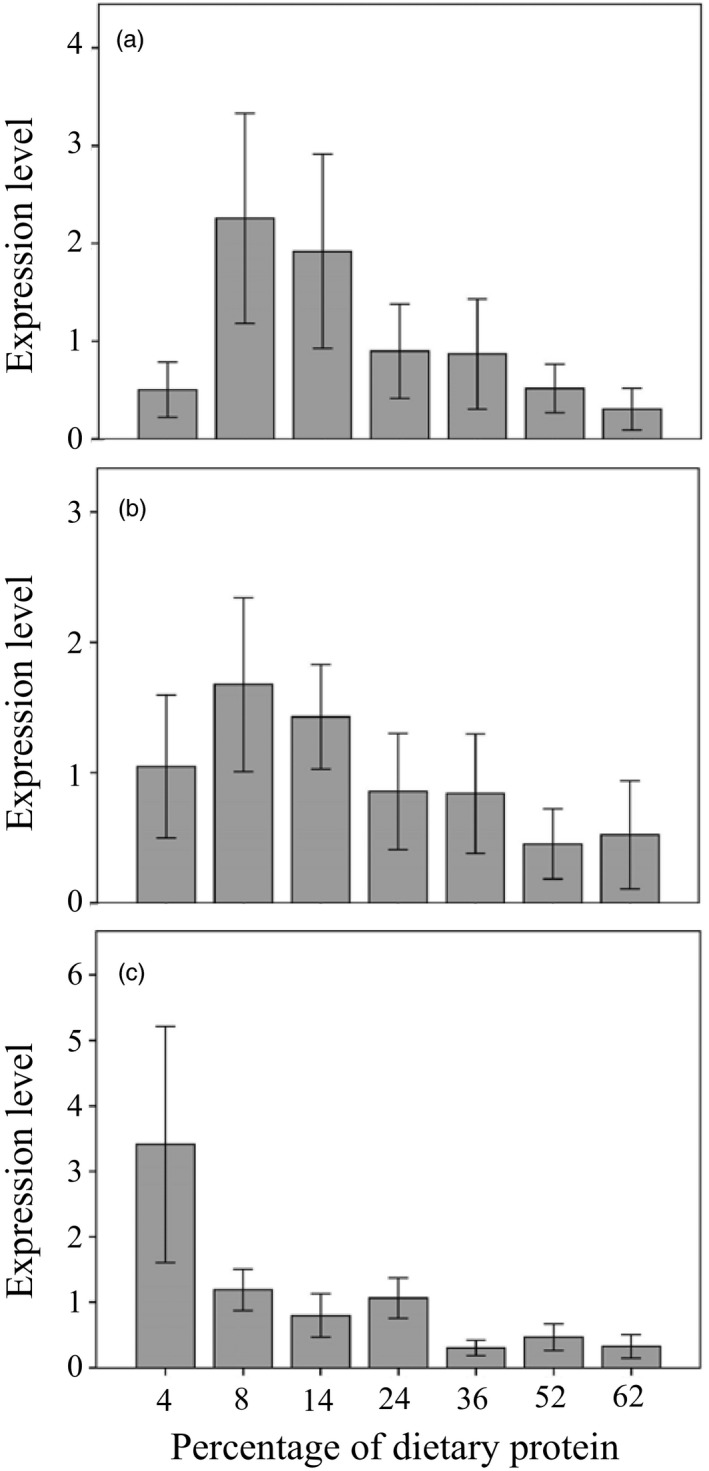
Expression levels of antimicrobial genes (mean ± *SE*) according to the percentage of protein in the diet at: (a) 25% mortality, (b) 50% mortality, (c) 75% mortality

Our data show that the expression levels of the genes coding for AMPs were significantly influenced by the P:C ratio in the diet (expression data for all AMPs; Kruskal–Wallis test: 25% mortality, *χ* ^2^ = 43.619, *df* = 6, *p* ≤ .001, *N* = 157; 50% mortality, *χ* ^2^ = 27.279, *df* = 6, *p* ≤ .001, *N* = 158; 75% mortality, *χ* ^2^ = 51.345, *df* = 6, *p* ≤ .001, *N* = 153; Figure 4). The expression level of the genes coding for AMPs was overall negatively associated with dietary P:C, and this was observed at the three sampling times, though there is some suggestion of nonlinear trends in the earlier sampling points (Figure 4). Expression of genes coding for immune receptors was significantly influenced by dietary P:C; however, we did not detect any clear pattern of variation (Figure S2 and Table S7). Diet composition did not influence expression levels of genes coding for molecules involved in the transduction of the immune signal (Table S7).

**Figure 5:**
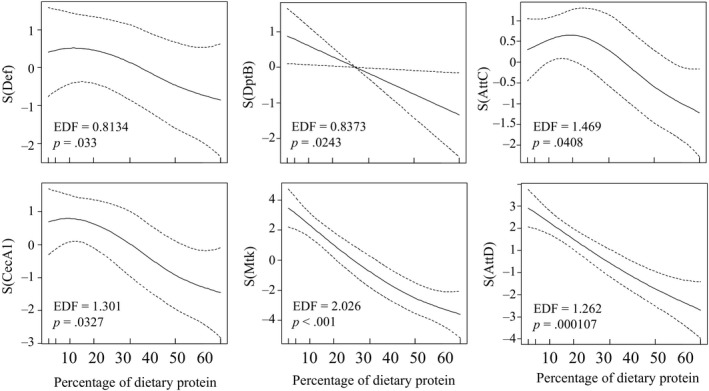
Estimated nonparametric smooths of antimicrobial gene expression levels from the generalized additive model according to the percentage of dietary protein at 50% survival [Def = Defensin (*n* = 19), DptB = DiptericinB (*n* = 18), AttC = AttacinC (*n* = 16), CecA1 = CecropinA1 (*n* = 18), Mtk = Metchnikowin (*n* = 16), AttD = AttacinD (*n* = 19)]

When we looked in more detail at the effect of dietary P:C on the expression levels of the specific genes, we found significant negative nonlinear relationships between the level of expression and the percentage of dietary protein for six out of nine genes coding for antimicrobial peptides (Figure 5), which reveals that antimicrobial peptide expression is tightly linked with the macronutrient balance in the diet. This diet‐dependent effect on antimicrobial peptide expression was consistent throughout the flies' lifespan (see Figure S3).

Interestingly, we found that the effect of dietary P:C can vary depending on the sampling point and the specific gene. For example, for the pattern recognition proteins, gene expression was positively associated with P:C for PGRPSC2, GNBP1 (at 25% mortality only for both genes) and PGRPLC (at 50% mortality only), whereas there was a negative association for PGRPSA (at 25% and 75% mortality) and PGRPSB1 (at all sampling points) (see Figure S3). Expression of genes coding for proteins involved in the immune‐signal transduction (i.e. Dif, Imd, Relish, Thor, Toll, Spätzle) was generally not significantly influenced by dietary P:C (Figure S3). Together, these results suggest that a carbohydrate‐biased diet can maintain a higher constitutive expression of antimicrobial peptide genes that might allow flies to better fight infections and injuries.

## 4 DISCUSSION

Our results confirm the key role of protein and carbohydrate in immunity and resistance to infection. Although the dietary ratio of protein to carbohydrate (P:C) modulated the expression of genes linked to innate immunity, it did not affect all immune molecules in the same way (see also Cotter et al., 2019; Cotter, Simpson, et al., 2011). While AMP expression levels were overall negatively affected by the relative amount of protein in the diet, the effects of dietary P:C on molecules involved in the recognition of pathogens depended on gene identity. In addition, overall, no effect of dietary P:C was detected on molecules involved in the transduction of the immune signal.

In a diet‐choice experiment, we showed that flies infected with *M. luteus* decreased their protein consumption while maintaining carbohydrate intake at the same level as non‐infected individuals. Anorexia – that is a sharp decreased in overall food intake – has been proposed to enhance tolerance and/or resistance [see, for instance, Ayres and Schneider (2009) and Adamo, Bartlett, Le, Spencer, and Sullivan (2010)]. Our results strongly support the notion that a specific decrease in protein intake, rather than overall food, may underpin this effect (Cotter, Simpson, et al., 2011; see also Fontana and Partridge (2015) for a similar discussion on the effects of nutrients on longevity). Similar results were recently observed in the true fruit fly *Bactrocera tryoni* (Dinh, Mendez, Tabrizi, & Ponton, 2019). Furthermore, while sham‐infected individuals survived better on low P:C, we did not observe any significant shift in their diet choice. The magnitude of the nutritional effects in sham‐infected flies was smaller than that of infected flies, which might explain these results.

The diversity of immune responses during infections and repair mechanisms following injuries might be influenced differently by the host's physiology and nutrition. Ayres and Schneider (2009) observed that the effects of food dilution on the outcome of infection depended on the strain of bacteria used to infect flies. Furthermore, because in most studies, food quality was manipulated through a decrease of the total nutritional content, it is difficult to conclude if the positive effect of the lack of one nutrient can be outweighed by the negative effect of the lack of another nutrient. In our experiment, infected flies maintained their carbohydrate intake while decreasing protein intake. The positive effects of this shift in diet composition might have not been observed if flies were restricted to a diet where both nutrient concentrations were simultaneously decreased. More investigations are nevertheless needed to fully understand the separate and combined effects of macronutrients on immune pathways when individuals are infected by different types of parasites.

Interestingly, the effects of dietary manipulation on immunity and resistance might be dependent on the developmental stage of the insect. Several experiments have shown that caterpillars (i.e. lepidopteran larvae) on a high‐protein diet are more resistant than larvae on a low‐protein diet, which contrasts with our results in an adult dipteran (Lee et al., 2006; Povey et al., 2009, 2014). Also, the manipulation of diet at an early developmental stage might affect immunity later in life. For instance, Fellous and Lazzaro (2010) have shown that nutrition at the larval stage influences immunity in the adult stage. More particularly, an increase in yeast (protein) supply to *D. melanogaster* larvae resulted in adults with greater immune gene expression while larval immunity was not affected. What drives differences in the interactions between diet and resistance to infection through development stages still needs to be fully explored, but actively growing juveniles can reasonably be assumed to have greater protein requirements than adults.

The cross‐regulation between immune and metabolic pathways involves molecules such as Forkhead box O (FOXO), target of rapamycin (TOR) and 5′ AMP‐activated protein kinase (AMPK) in mammals and *Drosophila* (Abdel‐Nour, Tsalikis, Kleinman, & Girardin, 2014; Becker et al., 2010; Lee, Rayyan, Liao, Edery, & Pletcher, 2017 ;Martin et al., 2012; Seiler et al., 2013). Inhibition of TOR signalling has been shown to promote a pro‐autophagic and inflammatory environment that is essential for clearing infections (Chakrabarti, Liehl, Buchon, & Lemaitre, 2012; Martin et al., 2012), which might result from nutrient deficiencies, such as amino acid deprivation following host membrane damage (Tattoli et al., 2012). Varma et al. (2014) have also shown that inhibiting the TOR pathway using mutants and the drug rapamycin results in an enhanced expression of several AMPs in *Drosophila* (Varma et al., 2014). Interestingly, this system can be manipulated by pathogens that have evolved ways to maintain TOR complex activity in an amino acid‐independent manner (Clippinger, Maguire, & Alwine, 2011). Transcription factors, such as FOXO, that interplay with metabolic pathways can activate the expression of AMPs independently of the NF‐kB‐derived innate immune pathways (Becker et al., 2010). This might explain why in our experiment we found different transcription levels for AMPs under the different dietary regimes without an increase in the expression level of genes from the intracellular immune signalling pathways. Furthermore, a recent study in *Spodoptera* caterpillars found that the relationship between the expression of immune genes and the activity of the expressed protein was strongly influenced by the P:C ratio of the diet, suggesting influences of nutrient availability at several stages throughout the transcription–translation pathway (Cotter et al., 2019).

Dietary protein‐to‐carbohydrate ratio was predicted to modulate TOR activation, as shown in mice (Solon‐Biet et al., 2014). As a result, we predicted that antimicrobial peptides are upregulated on high‐carbohydrate, low‐protein diets because of low TOR activation (see also Lee et al., 2017; Varma et al., 2014). However, we did not detect a prophylactic effect of carbohydrate per se, as it has been suggested in an earlier study (Galenza et al., 2016). Interestingly, Bajgar et al. (2015) have shown that when fly larvae are infected with a parasitoid, there is a metabolic switch that leads to a breakdown of energy storage compounds, glycogen and tryacylglycerol, with an increase in the level of glucose in the haemolymph. In parallel, less dietary glucose is incorporated into proteins, while immune cells increase their glucose intake and help the host to better fight the infection. This physiological mechanism could lead infected individuals to ingest a diet biased for carbohydrate, since they would require carbohydrate, more than protein, to fuel their immune response. However, when flies are fed an excess of glucose for a few generations before infection, they resist infections less well (i.e. greater pathogen load) than when fed a low glucose diet (Unckless, Rottschaefer, & Lazzaro, 2015). Comparing the effects of macronutrients on immunity and resistance through multiple generations would be a fruitful continuation to this work. Furthermore, better understanding how metabolic state before infection influences immune responses would give insights into the interactions between metabolic disease and resistance to infections. Micronutrients are also important food components that can modulate immunity (e.g. Calder & Kulkarni, 2017). We here approached foods as mixtures of macronutrients (and correlated content of micronutrients) and do not specifically address the effects of micronutrients on fly immunity. More investigations through specific manipulations of dietary micronutrient content would allow to further explore the role of micronutrients on immunity and resistance to infection to be explored.

Our results show that the modulation in macronutrient intake observed in flies injected with *M. luteus* decreased differences in survival between infected and control flies 15 days after infection. Self‐medication has been traditionally seen as animals using molecules such as secondary plant compounds or other non‐nutritive substances with antiparasitic activity (Raubenheimer & Simpson, 2009; de Roode, Lefevre, & Hunter, 2013). However, our work reinforces the idea that self‐medication can happen through modulating macronutrient selection to stimulate the immune response and potentially compensate for the negative effects of the infection on fitness traits (see, for instance, Abbott, 2014; Bashir‐Tanoli & Tinsley, 2014; Galenza et al., 2016; Povey et al., 2014). In this experiment, it is, however, difficult to assess the direct effects of macronutrients on immunity. It has been previously shown that flies restricted to low P:C diets have a lower fecundity than flies fed higher P:C diets (Fanson, Weldon, Perez‐Staples, Simpson, & Taylor, 2009; Lee et al., 2008). Pleiotropic mechanisms that may regulate allocation of resource between reproductive and immune processes have been suggested in insects (Schwenke, Lazzaro, & Wolfner, 2016) and a shift in P:C ratio might modulate the trade‐off between reproduction and immunity, limiting reproduction to activate immunity. Life‐history strategies are often state‐dependent (McNamara & Houston, 1996); infection has often been mooted as a trigger for terminal investment (e.g. Velando, Drummond, & Torres, 2006), but this may depend on the severity of the infection, the risk of death and an animal's residual reproductive value (Cotter, Ward, & Kilner, 2011). Shifting to a low P:C diet during infection could therefore represent a form of reproductive restraint, withholding resources from growth and reproduction to increase longevity (McNamara, Houston, Barta, Scheuerlein, & Fromhage, 2009). The interplay between nutritional ecology, host–parasite interactions and state‐dependent life history theory would be an interesting focus for future research.

## AUTHORS' CONTRIBUTIONS

F.P. and S.J.S. designed the experiments. F.P., K.R. and S.S.K. ran the experiments. F.P. and J.M. analysed the results. F.P., J.M., K.R., S.C.C., S.S.K., K.W. and S.J.S. wrote the manuscript.

## Data Availability

Data available from the Dryad Digital Repository: https://doi.org/10.5061/dryad.dv41ns1td (Ponton, 2020).
