# Supplementary material for: Macronutrients modulate survival to infection and immunity in Drosophila
Source: J Anim Ecol. 2019 Dec 9;89(2):460–70. doi: 10.1111/1365-2656.13126 (PMC7027473; doi:10.1111/1365-2656.13126)

Supplementary Figure 1. Life expectancy Kaplan–Meier curves with 95% confidence for flies fed seven diets varying in the percentage of protein (P) (Log Rank test, χ^2=3166.5, df=6, p≤0.001).

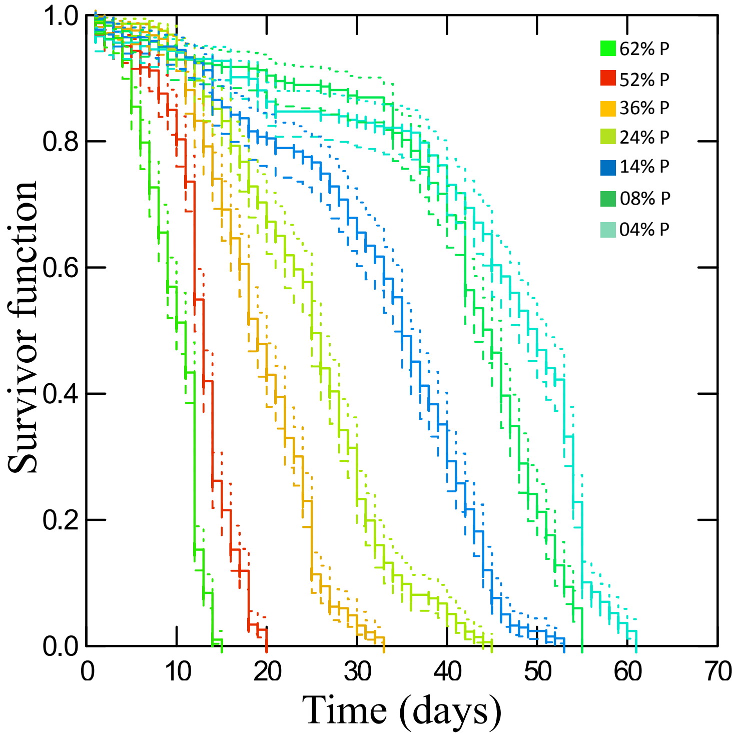

Supplementary Figure 2. Expression levels of immune receptors (mean±SE) according to the percentage of protein in the diet at: A. 25% mortality, B. 50% mortality, C. 75% mortality.

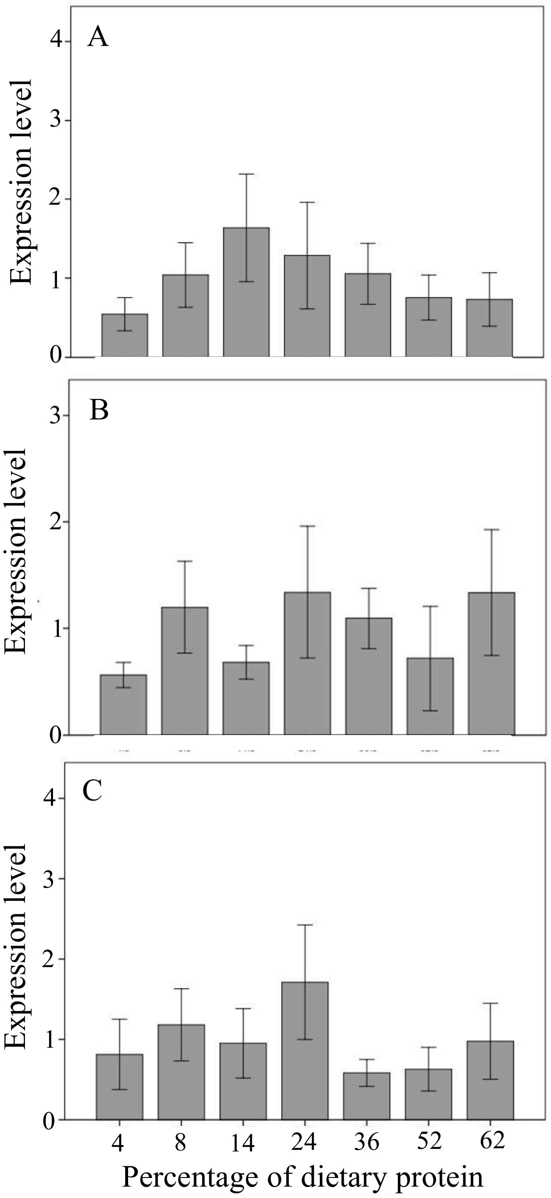

Supplementary Figure 3. Estimated nonparametric smooths of immune gene expression levels from the generalized additive model according to the percentage of dietary protein at 25%, 50% and 75% mortality.

Supplementary Figure 3 (1/4) Molecules involved in the recognition of pathogens

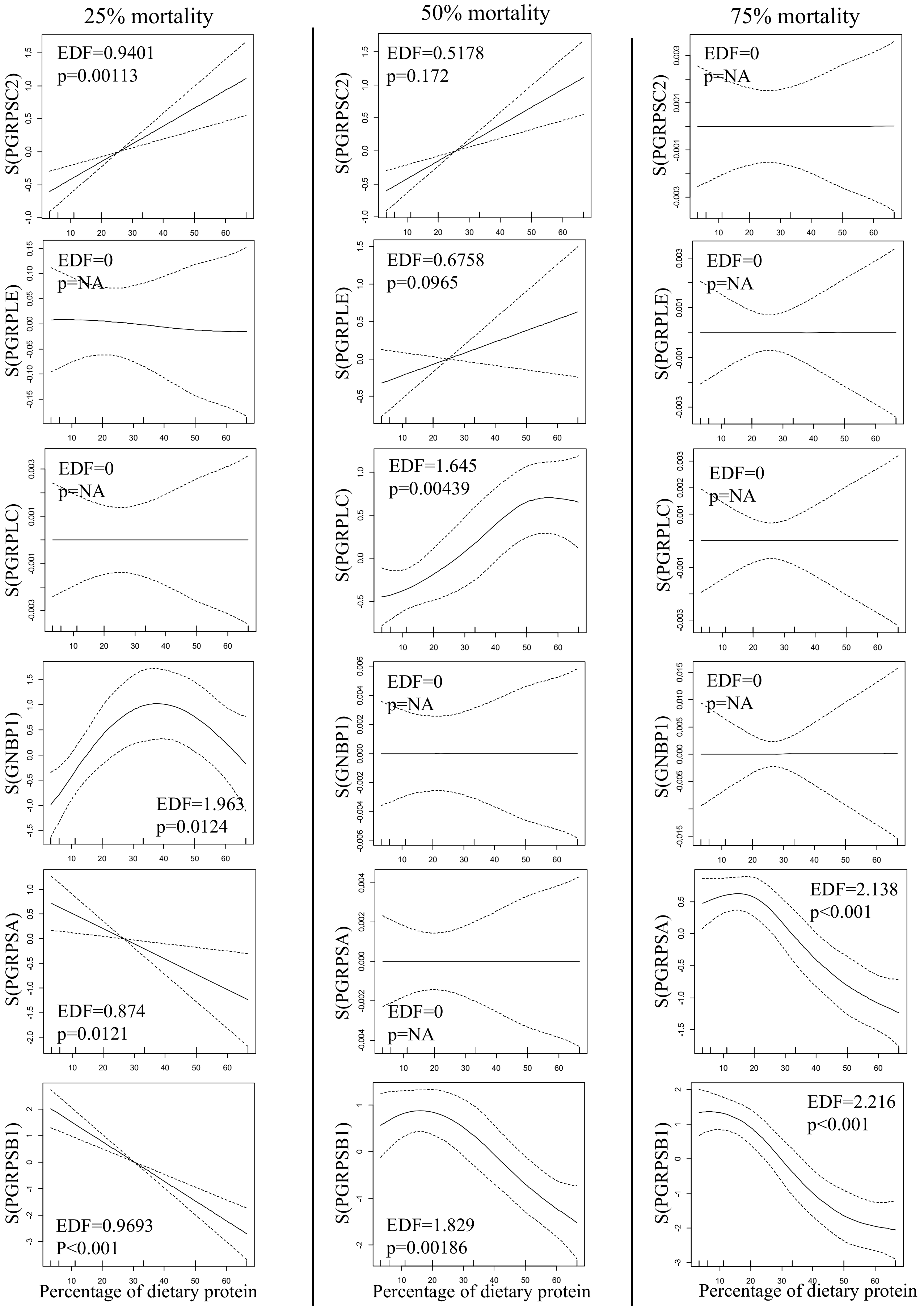

Supplementary Figure 3 (2/4) Molecules involved in the transduction of the immune signal

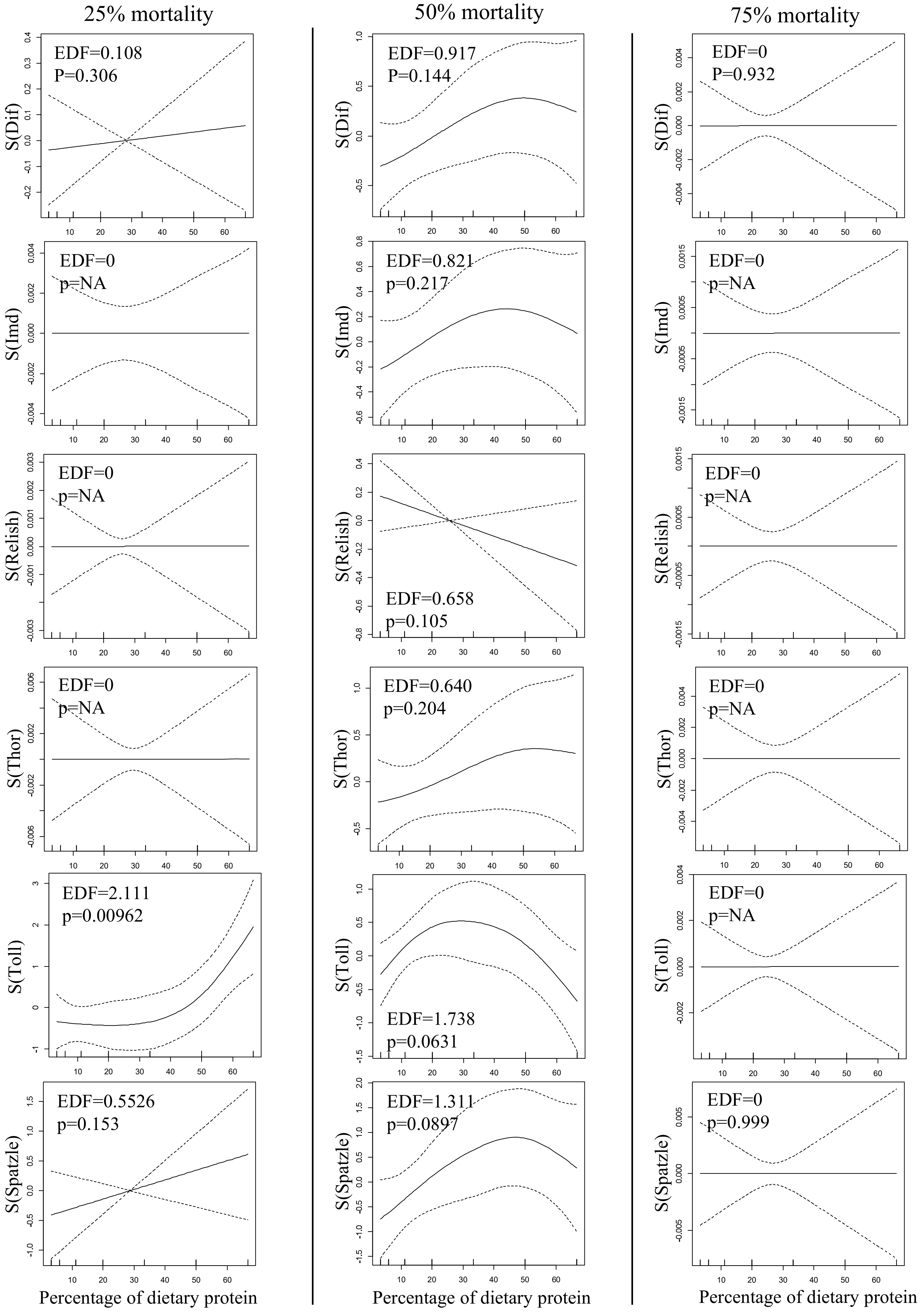

Supplementary Figure 3 (3/4) Antimicrobials

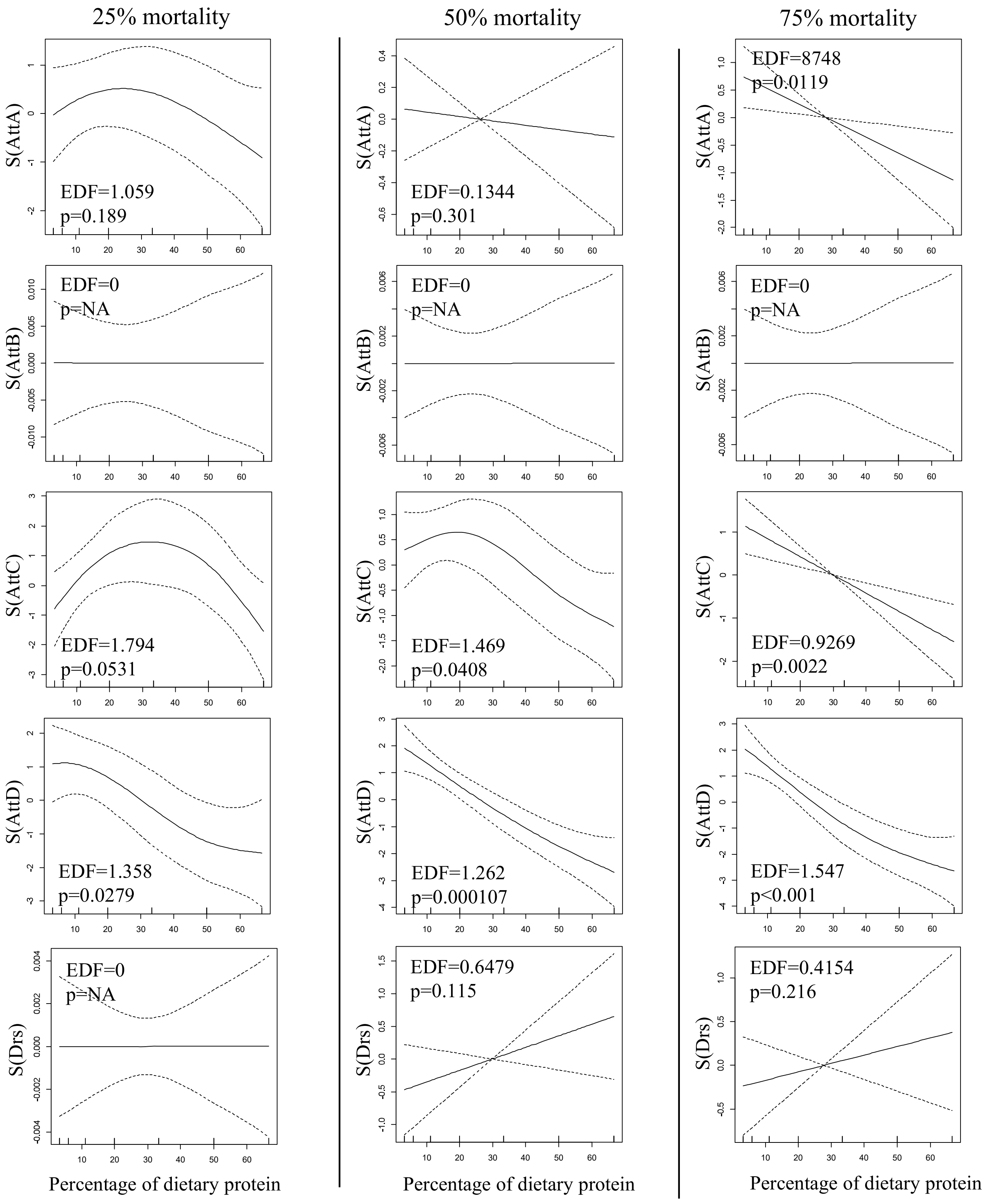

Supplementary Figure 3 (4/4) Antimicrobials

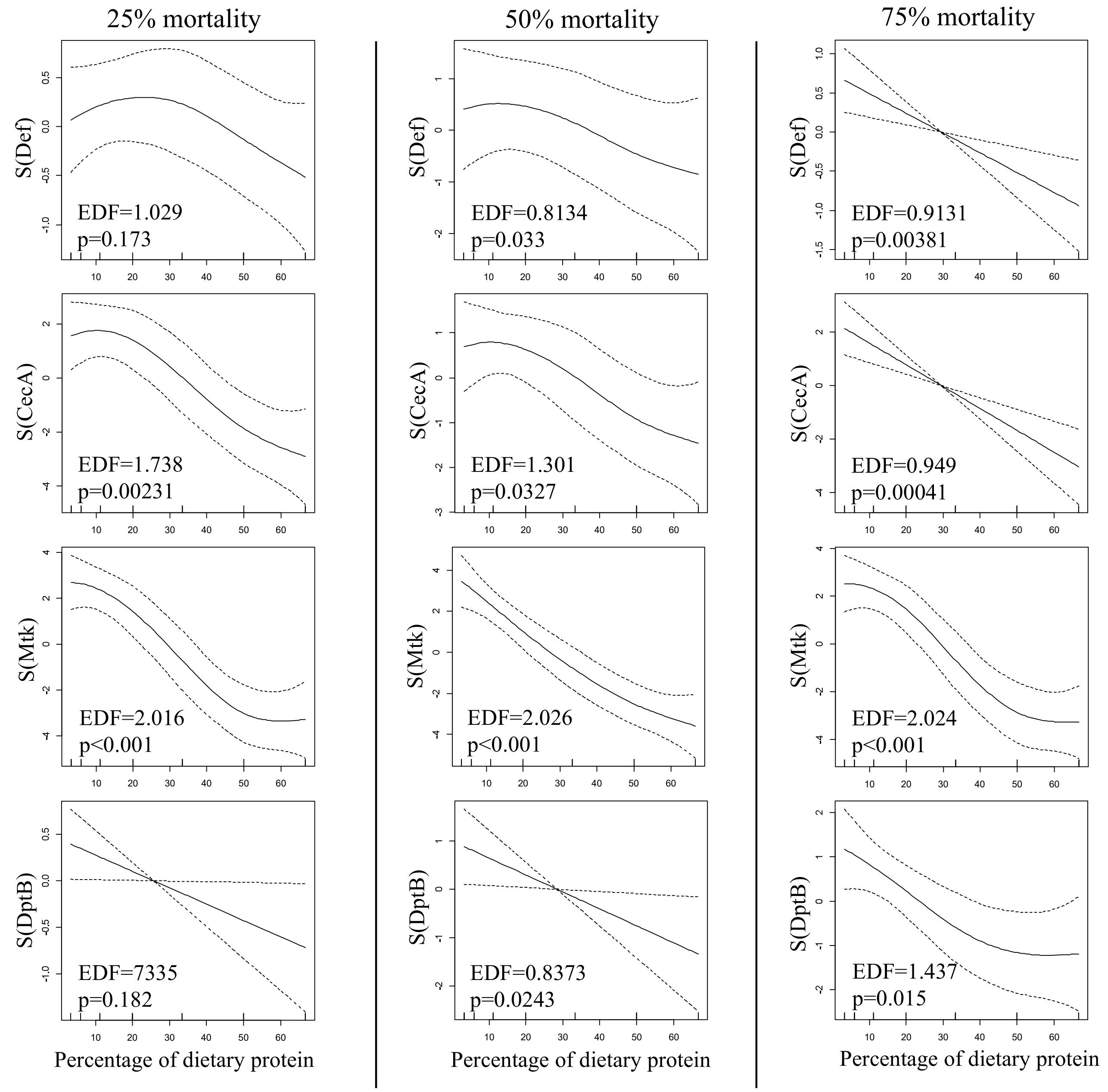

Supplement: Supplementary file 1 [file JANE-89-460-s001.pdf]
