# Supplementary material for: Macronutrients modulate survival to infection and immunity in Drosophila
Source: J Anim Ecol. 2019 Dec 9;89(2):460–70. doi: 10.1111/1365-2656.13126 (PMC7027473; doi:10.1111/1365-2656.13126)
Supplement: Supplementary file 2 [file JANE-89-460-s002.docx]

**Supplementary Table S1.** Primers of genes used in the RT-qPCR experiment.

| **Gram-negative bacteria binding protein 2 (GNBP2, CG4144)** | | **Source of primer sequences** | |
| --- | --- | --- | --- |
| Primer forward | GGATTTAGCGTTTCCATACCG | This study | |
| Primer reverse | GAAGGATGTGAAATTCCGATTG |  |  |
| **Peptidoglycan recognition protein SA (PGRP-SA, CG11709)** | | | |
| Primer forward | GCGATCAGGGCGTAATCCT | | This study |
| Primer reverse | CTCTGGGTGCTGATCACCTG | |  |
| **Spatzle (Spz, CG6134)** | | | |
| Primer forward | ATATCGCGGCATTTCATCAG | | This study |
| Primer reverse | CACGTTTGCGAGACACACAG | |  |
| **Dorsal-related immunity factor (Dif, CG6794)** | | | |
| Primer forward | TCTGTCTGACCCAGTGCATTC | | This study |
| Primer reverse | TATATCGCCGAAAGCCTCCT | |  |
| **Attacin-A (AttA, CG10146)** | | | |
| Primer forward | GGCGGAACTTTGGCCTAC | | This study |
| Primer reverse | AGATTGTGTCTGCCATTGTTGA | |  |
| **Cecropin A1 (CecA1, CG1365)** | | | |
| Primer forward | TTTCGTCGCTCTCATTCTGG | | Zambon et al. (2005) PNAS 102: 7257-7262. |
| Primer reverse | GACAATCCCACCCAGCTTCCCGATTG | |  |
| **Cecropin C (CecC, CG1373 )** | | | |
| Primer forward | TCATCCTGGCCATCAGCATT | | Becker et *al.* (2010) Nature 463: 369-373. |
| Primer reverse | CGCAATTCCCAGTCCTTGAAT | |  |
| **Diptericin B (DptB, CG10794)** | | | |
| Primer forward | ACTGGCATATGCTCCCAATTTT | | This study |
| Primer reverse | CTCAGATCGAATCCTTGCTTTG | |  |
| **Defensin (Def, CG1385)** | | | |
| Primer forward | CCACATGCGACCTACTCTCCA | | Zambon *et al.* (2005) PNAS 102: 7257-7262. |
| Primer reverse | GACAAGAACGCAGACGGCCTTG | |  |
| **Metchnikowin (Mtk, CG8175)** | | | |
| Primer forward | CCACCGAGCTAAGATGCAA | | Steckel and Boutros (2005) Biochemica 3: 17-19. |
| Primer reverse | TCTGCCAGCACTGATGTAGC | |  |

**Supplementary Table S2.** Sampling at 25%, 50% and 75% mortality on the life expectancy curves for each of the seven diets varying in the protein-to-carbohydrate ratio.

| **% Protein** | **25% mortality** | | **50% mortality** | | **75% mortality** | |
| --- | --- | --- | --- | --- | --- | --- |
|  | average (days) | SD | average (days) | SD | average (days) | SD |
| 4 | 35.67 | 13.65 | 49.00 | 5.29 | 55.00 | 2.00 |
| 8 | 40.33 | 5.69 | 45.67 | 5.51 | 48.00 | 6.00 |
| 14 | 25.67 | 10.69 | 35.67 | 4.62 | 39.67 | 4.04 |
| 24 | 17.67 | 4.51 | 25.67 | 4.04 | 30.67 | 3.21 |
| 36 | 16.00 | 4.00 | 19.33 | 4.51 | 21.67 | 3.51 |
| 52 | 11.33 | 1.15 | 13.33 | 1.15 | 15.67 | 2.52 |
| 62 | 7.67 | 2.52 | 10.00 | 2.65 | 11.67 | 0.58 |

**Supplementary Table S3.** List of genes used in the design of the TLDA card assay.

| **Annotation** | **Symbol** | **Name** | **Functional group** |
| --- | --- | --- | --- |
| CG4432 | PGRP-LC | Peptidoglycan recognition protein LC | Pathogens recognition |
| CG9681 | PGRP-SB | Peptidoglycan recognition protein SB | Pathogens recognition |
| CG8995 | PGRP-LE | Peptidoglycan recognition protein LE | Pathogens recognition |
| CG11709 | PGRP-SA | Peptidoglycan recognition protein SA | Pathogens recognition |
| CG14745 | PGRP-SC2 | Peptidoglycan recognition protein SC2 | Pathogens recognition |
| CG6895 | GNBP1 | Gram-negative bacteria binding protein 1 | Pathogens recognition |
| CG8846 | Thor | Thor/4E-BP | Transduction of the immune signal |
| CG5490 | Tl | Toll | Transduction of the immune signal |
| CG5576 | Imd | Immune deficiency | Transduction of the immune signal |
| CG6134 | Spz | Spatzle | Transduction of the immune signal |
| CG1385 | Def | Defensin | AMP |
| CG1365 | CecA1 | Cecropin A1 | AMP |
| CG8175 | Mtk | Metchnikowin | AMP |
| CG10810 | Drs | Drosomycin | AMP |
| CG10794 | DptB | Diptericin B | AMP |
| CG6794 | Dif | Dorsal-related immunity factor | AMP |
| CG11992 | Rel | Relish | AMP |
| CG10146 | AttA | Attacin A | AMP |
| CG18372 | AttB | Attacin B | AMP |
| CG4740 | AttC | Attacin C | AMP |
| CG7629 | AttD | Attacin D | AMP |
| CG1873 | Ef1α100E | Elongation factor 1α100E | Reference gene |
| CG1913 | αTub84B | α-Tubulin at 84B | Reference gene |
| CG7939 | RpL32 | Ribosomal protein L32 | Reference gene |
| FBgn0061475 | 18SrNA | 18SrNA | Reference gene |

**Supplementary Table S4.** Macronutrient intakes for flies in the food choice experiment following three treatments (i.e., Control, *M. luteus*- and Sham -infected).

| Treatment | Total carbohydrate eaten (µg) | SD | Total protein eaten (µg) | SD |
| --- | --- | --- | --- | --- |
| Naïve | 534.47 | 159.15 | 136.35 | 82.76 |
| *M. luteus* | 532.81 | 123.01 | 50.83 | 22.85 |
| Sham | 448.46 | 100.70 | 140.41 | 76.08 |

**Supplementary Table S5. A**. Generalized linear model (Binomial error distribution) analyses to test for the effects of diet (low, medium and high P:C ratio) and treatment (*M. luteus*- and sham-infected, and naïve) on the number of dead flies after 15 days. **B**. Percentage of dead flies in each treatment and diet after 15 days.

**A**

| **Factors** | **df** | **df residuals** | **Residuals deviance** | **p** |
| --- | --- | --- | --- | --- |
| Diet | 2 | 1328 | 1260.2 | **<0.001** |
| Treatment | 2 | 1326 | 1213.8 | **<0.001** |
| Diet X Treatment | 4 | 1322 | 1191 | **<0.001** |

**B**

| **Diet** | **Treatment** | **Percentage of dead flies** |
| --- | --- | --- |
| High P:C | Naïve | 97.04 |
| High P:C | Sham | 99.28 |
| High P:C | *M. luteus* | 94.56 |
| Medium P:C | Naïve | 21.79 |
| Medium P:C | Sham | 52.98 |
| Medium P:C | *M. luteus* | 62.59 |
| Low P:C | Naïve | 19.31 |
| Low P:C | Sham | 26.35 |
| Low P:C | *M. luteus* | 29.25 |

**Supplementary Table S6.** Gene details and statistical analyses following RT-qPCR assays.

| **Target gene** | **Full name** | **One-way ANOVA** | **N** |
| --- | --- | --- | --- |
| GNBP2 | Gram-negative bacteria binding protein 2 | F_2,10_= 0.062; p=0.941 | Sham=4; *M. luteus*=3 |
| PGRPSA | Peptidoglycan recognition protein SA | F_2,12_= 1.321; p=0.314 | Sham=4; *M. luteus*=3 |
| spz | Spatzle | F_2,14_= 42.531; **p<0.001** | Sham=5; *M. luteus*=4 |
| Dif | Dorsal-related immunity factor | F_2,12_= 7.546; **p=0.012** | Sham=4; *M. luteus*=3 |
| AttA | Attacin A | F_2,9_= 37.996; **p<0.001** | Sham=4; *M. luteus*=3 |
| CecA1 | Cecropin A1 | F_2,12_= 36.365; **p<0.001** | Sham=5; *M. luteus*=4 |
| CecC | Cecropin C | F_2,13_=67.678; **p<0.001** | Sham=5; *M. luteus*=4 |
| DptB | Diptericin B | F_2,14_= 46.543; **p<0.001** | Sham=5; *M. luteus*=4 |
| Def | Defensin | F_2,14_= 29.390; **p<0.001** | Sham=5; *M. luteus*=4 |
| Mtk | Metchnikowin | F_2,14_=30.046; **p<0.001** | Sham=5; *M. luteus*=4 |
|  |  |  |  |

**Supplementary Table S7.** Kruskal-Wallis analyses to test for the effect of the percentage of dietary protein on the level of expression of immune receptors genes and genes coding for molecules involved in the transduction of the immune signal.

| **Gene class** | χ^2^ | df | *p* | N |
| --- | --- | --- | --- | --- |
|  |  |  |  |  |
| **25% mortality** |  |  |  |  |
| *Immune receptors* | 16.599 | 6 | **0.011** | 105 |
| *Transduction immune signal* | 12.653 | 6 | **0.049** | 126 |
| **50% mortality** |  |  |  |  |
| *Immune receptors* | 13.810 | 6 | **0.032** | 110 |
| *Transduction immune signal* | 11.097 | 6 | 0.085 | 134 |
| **75% mortality** |  |  |  |  |
| *Immune receptors* | 19.336 | 6 | **0.004** | 108 |
| *Transduction immune signal* | 5.944 | 6 | 0.429 | 125 |
